# Sociodemographic and clinical predictors of delay to and length of stay with early intervention for psychosis service: findings from the CRIS-FEP study

**DOI:** 10.1007/s00127-023-02522-z

**Published:** 2023-06-23

**Authors:** Sherifat Oduola, Tom K. J. Craig, Eduardo Iacoponi, Alastair Macdonald, Craig Morgan

**Affiliations:** 1 School of Health Sciences, University of East Anglia, Norwich Research Park, Norwich, NR4 7TJ UK; 2 Department of Health Service and Population Research, Institute of Psychiatry, Psychology & Neuroscience, King’s College London, De Crespigny Park, Denmark Hill, London, SE5 8AF UK; 3 South London & Maudsley NHS Foundation Trust, Denmark Hill, London, SE5 8AZ UK

**Keywords:** Early intervention psychosis, Treatment delays, First episode psychosis, Pathways to care, Length of stay

## Abstract

**Purpose:**

We investigated the influence of sociodemographic and clinical characteristics on delay to early intervention service (EIS) and the length of stay (LOS) with EIS.

**Methods:**

We used incidence data linked to the Clinical Record Interactive Search—First Episode Psychosis (CRIS-FEP) study. We followed the patients from May 2010 to March 2016. We performed multivariable Cox regression to estimate hazard ratios of delay to EIS. Negative binomial regression was used to determine LOS with EIS by sociodemographic and clinical characteristics, controlling for confounders.

**Results:**

343 patients were eligible for an EIS, 34.1% of whom did not receive the service. Overall, the median delay to EIS was 120 days (IQR; 15–1668); and the median LOS was 130.5 days (IQR 0–663). We found that women (adj.HR 0.58; 95%C I 0.42–0.78), living alone (adj.HR: 0.63; 95% CI 0.43–0.92) and ethnicity (‘Other’: adj.HR 0.47; 95% CI 0.23–0.98) were associated with prolonged delay to EIS. However, family involvement in help-seeking for psychosis (adj.HR 1.37; 95% CI 1.01–1.85) was strongly associated with a shorter delay to EIS. Patients who have used mental health services previously also experienced long delays to EIS.

**Conclusions:**

Our analyses highlight the link between sociodemographic status, help-seeking behaviours, and delay to EIS. Our findings also show the vulnerability faced by those with a previous mental health problem who later develop psychosis in receiving specialist treatment for psychosis. Initiatives that ameliorate indicators of social disadvantage are urgently needed to reduce health inequalities and improve clinical outcomes.

**Supplementary Information:**

The online version contains supplementary material available at 10.1007/s00127-023-02522-z.

## Introduction

First episode psychosis often begins with a prodrome phase of a low threshold of symptoms and altered functioning before the onset of frank psychosis. The onset of psychosis typically occurs when people are young, and they may be reluctant to seek help because of the blur between psychotic symptoms and normal developmental changes [1, 2]. Timely access to specialist early intervention for psychosis has been shown to halt poorer outcomes [3-5]. Therefore, improving the short- and long-term outcomes of psychosis has been the preoccupation of service providers globally [5, 6]. Since the late 1990s and 2000s, early intervention services for psychosis have been established in many high- and middle-income countries[7-9]. Several of these have been carefully evaluated and showed that early intervention for psychosis care is superior in improving clinical and functional outcomes compared with standard care [10-14]. Some studies have highlighted individual, clinical, and service-related factors impeding access to EI service [15, 16]. For example, living alone, unemployment and social isolation are linked to a longer duration of untreated psychosis [16]. In terms of pathways to care, ethnicity is well documented as a risk for involuntary admissions [17] and criminal justice system involvement [18].

To establish parity of esteem between physical and mental health, the UK government introduced the Access and Waiting Time Standard for early intervention for psychosis services in England [19]. It recommended that adults presenting with a first episode of psychosis (FEP) should start treatment in early intervention for psychosis services within 2 weeks of referral [20]. However, despite the widely documented evidence that early initiation of treatment in early intervention for psychosis service improves longer term outcomes, the optimal duration of stay with early intervention service (EIS) has been a matter of ongoing debate. Only a handful of studies have been conducted to investigate the ideal period of stay and treatment with an EIS [21, 22].

To date, much of our understanding of the factors associated with delay to an EIS has been gleaned through the lens of the duration of untreated psychosis [16, 23, 24], which considers the time between the onset of symptoms and first contact with a mental health service or first antipsychotic treatment; therefore, our understanding specifically in delay in reaching an EIS is distorted. Hence, high-quality research on the influence of sociodemographic, clinical, and pathways to care characteristics on delay to reaching an EIS and, subsequently how long patients stay with an EIS is limited. A better understanding of factors associated with delay in reaching an early intervention for psychosis service will inform the development of strategies to ameliorate them. From the few available studies that focus on pathways to EIS, several are based on cross-sectional samples and do not account for the non-randomness of the length of the pathway to EIS [5, 15]. To our knowledge, there has not been a longitudinal cohort study that investigated the associations between sociodemographic clinical characteristics, length of delay to EIS, and length of stay with EIS. To address these gaps, in this study, we use an epidemiologically derived cohort of first episode psychosis patients. We sought to (a) estimate the length of delay to an EIS from first contact for psychosis, (b) examine sociodemographic, pathways to care, and clinical factors associated with delay to EIS, and (c) determine the length of stay with EIS and the associated factors.

## Methods

### Samples

The study was conducted in two inner city areas of London, served by the South London and Maudsley NHS Foundation Trust (SLaM). These are the London boroughs of Lambeth and Southwark, with a combined population of 625,300 people [25]

### Inclusion/exclusion criteria

The present study is part of a larger incidence study conducted between May 2010 and April 2012 [26]. We included participants if they were residents in the London boroughs of Lambeth or Southwark, (b) aged 18–64 years old (inclusive) at presentation, (c) with a clinical diagnosis of a psychotic disorder (i.e., ICD F20-29, F30-33), and (d) were in first contact with mental health services for psychosis. Exclusion criteria were: (a) evidence of psychotic symptoms with an organic cause, (b) transient psychotic symptoms resulting from acute intoxication, and (c) previous contact with services for psychotic symptoms.

At the time of this study, early intervention for psychosis services at SLaM typically offered a 3-year duration of treatment and support. The age eligibility criterion for accessing an EIS in SLaM was 18–35 years; this was before the introduction of the Access and Waiting Time Standard, i.e., 1 April 2016, when the upper age limit was extended to 65 year. Therefore, we restricted our analyses to those that met the earlier age (i.e. 18–35 years) criterion for an EIS.

### Study design, setting, and participants

The participants included in this study were drawn from an incidence cohort of patients with first episode psychosis (i.e., ICD F20-29, F30-33) assembled for the Clinical Record Interactive Search—First Episode Psychosis (CRIS-FEP) study[26]. In brief, we identified all patients presenting to the South London and Maudsley NHS Trust adult mental health services in Lambeth and Southwark for the first time with a psychotic disorder between May 2010 and April 2012. We used the South London and Maudsley NHS Trust (SLaM) Clinical Records Interactive Search (CRIS) system [27], which provides fully de-identified access to all SLaM electronic clinical records.

### Outcome variables and covariates

The primary outcomes were:

1. Time to acceptance by an early intervention service, measured from the date of the first presentation for psychosis or the discharge date from inpatient admission for psychosis (if admitted at first presentation). This time is considered to represent the beginning of delay to an EIS following a presentation for first episode psychosis in SLaM. Patients were followed until the date of acceptance to an EIS, end of the study (31 March 2016), or date of discharge from SLaM services, whichever came first.
2. Length of stay with EIS, measured from the date of acceptance to an EI service, and patients were followed until the end of the study (31 March 2016) or date of discharge from EI services or death, whichever came first. We considered this time to represent the start of the duration of time individuals received treatment from an EIS.

### Covariates

Sociodemographic, clinical, and pathways to care characteristics were collected as covariates: age, gender, ethnicity, living circumstances, employment status, duration of untreated psychosis, mode of onset of psychosis, and source of referral. Data on demographic and social circumstances were extracted from the patient’s de-identified electronic clinical records guided by the Medical Research Council Sociodemographic schedule MRC-SDS [28]. Ethnicity was self-ascribed and recorded in clinical records. We categorised ethnicity according to the 18 categories of the 2011 UK Census [29]. For the purpose of analysis, we collapsed the ethnic groups into seven categories in line with our previous studies [16, 26] as follows: white British, black Caribbean (black Caribbean and other black), black African, Asian (Indian, Pakistani, Bangladeshi, Chinese), white non-British (white Irish, white Gypsy, white Other), other (Arab, any other ethnic group) and mixed (all mixed groups).

Data relating to pathways to care, duration of untreated psychosis, and EIS encounters were also extracted from the patient’s de-identified electronic clinical records using the Personal and Psychiatric History Schedule (PPHS) [30]. Duration of untreated psychosis was measured from the date of onset of psychotic symptoms to the date of first contact with SLaM for psychosis [16].

### Statistical analysis

Stata (version 15) software was used to analyse the data [31]. Numbers, frequencies, mean, and medians, along with the standard deviation and interquartile range, were used as appropriate to describe the sample. Descriptive statistics for dependent and independent variables were obtained as median with interquartile range, with the two outcomes of delay to EIS and length of stay with EIS. Kaplan–Meier survival analysis and multivariable Cox regression were used to assess associations between delay to EIS and covariates. First, we performed univariable Cox regression for estimates of unadjusted hazard ratios for the delay to EIS, then adjusted for a-priori confounders (i.e., age, gender, ethnicity, living circumstances, and duration of untreated psychosis). The hazard ratios derived from Cox regression analyses represent the probability of receiving an EIS during the follow-up period. Therefore, a hazard ratio greater than 1 denotes an association of an independent variable with the shortest time to EIS.

To assess the association of independent variables with the length of stay (LOS) with EIS, we employed negative binominal regression, whilst taking into account the follow-up period using the *exposure* option in Stata for unadjusted and adjusted incidence rate ratios of LOS. Negative binomial regression models were used to overcome the over-dispersion of zero, and the data were not normally distributed (Pearson goodness-of-fit *X*^2^ = 1991.0, *df* = 342, *p* < 0.0001).

We addressed missing data in our multivariable regression analyses by including only patients with complete data on all variables included in the models. We conducted Bonferroni adjustments for multiple comparisons when relevant.

### Ethical approval

The CRIS system was approved as an anonymised dataset for secondary analysis by the Oxfordshire Research Ethics Committee (reference 08/H0606/71). We obtained local approval for this study via the CRIS Oversight Committee at the BRC South London and Maudsley NHS Foundation Trust (reference: 09–041).

## Results

Three hundred and forty-three patients aged 18–35 years were eligible for an EIS. The mean age was 26.1 (sd, 5.0) years, there were more men (n, 198 (57.7%)) than women, and black African patients (n, 95 (27.7%)) were the largest proportions of the sample. The median duration of untreated psychosis was 87 (IQR: 14–410) days; 142 (41.4%) patients were referred to mental health services via the Accident and Emergency department. Table 1 describes the study sample characteristics.

**Table 1:** Sociodemographic and pathways to care characteristics

Characteristics	Number in sample	% / SD/ IQR
Mean age (sd) years	26.1	5.0
Median DUP *in days (IQR)*	87	14- 410
Median EI delay *in days (IQR)*	120	15–1668
Length of stay with EI *in days (IQR)*	130.5	0–663
Gender		
Men	198	57.7
Women	145	42.3
Ethnicity		
White British	78	22.7
Black African	95	27.7
Black Caribbean	53	15.4
White non-British	40	11.7
Asian	30	8.7
Mixed	20	5.8
Other	27	7.9
Relationship status^1^		
Single	231	70.4
Married/Steady relationship	73	22.3
Divorced/Separated	24	7.3
Employment^2^		
Unemployed	203	63.4
Student	58	18.1
Employed	59	18.5
Lives with^3^		
Alone	74	22.2
Family/relatives	216	64.9
Other (e.g. hostel)	43	12.9
Source of referral		
GP referral	108	31.5
A&E referral	142	41.4
Police/Criminal Justice agency	50	14.6
Other (non-mental health professionals)	43	12.5
Involuntary admission		
No	252	73.9
Yes	89	26.1
Time of contact^4^		
Office hours	210	61.6
Out of hours	131	38.4
Family involvement in help-seeking		
No	207	60.7
Yes	134	39.3
Early intervention service received^2^		
Yes	222	65.1
No	119	34.9
Mode of contact		
Community	185	54.2
Inpatient	156	45.8
Mode of onset		
Acute (within a week)	83	24.3
Moderate (within a month)	61	17.9
Gradual (up to 6 months)	77	22.6
Insidious (more than 6 months)	120	35.2
Previous psychiatric service use^6^		
No	270	79.2
Yes	71	20.8

Missing data- 1 = 15 patients; 2 = 23 patients; 3 = 8 patients; 4 = 5 patients; 5 = 2 patients; 6 = 2patients

### Delay to EIS by sociodemographic characteristics

**Fig. 1:**
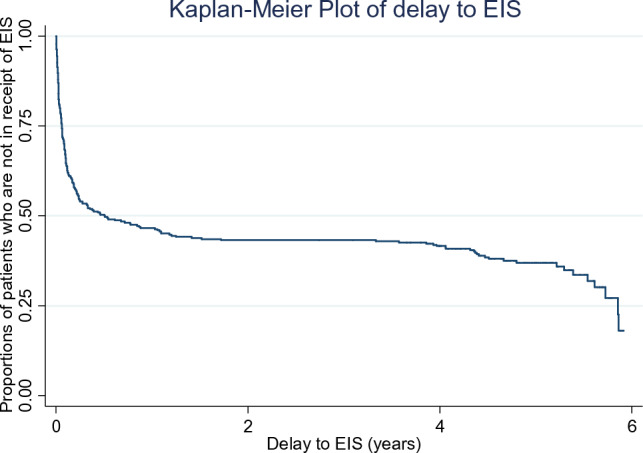
Kaplan–Meier plot of delay to an early intervention service

During the follow-up period, 318 patients with complete data constituted 701.9 person-years at risk, of whom 222 received EIS, meaning 34.9% of the eligible patients did not receive an EIS. The median delay to EIS was 120 (IQR; 15–1668) days. Kaplan–Meier plot (Fig. 1) shows the distribution of delay to EIS overtime. Table 2 presents the relationships between delay to EIS and sociodemographic characteristics. We found strong evidence of delay to EIS in older patients (adj.HR = 0.70; 95% CI = 0.52–0.94), among women (adj. HR = 0.60; 95% CI 0.44–0.80) and patients of ‘other’ ethnic groups (adj. HR = 0.51; 95% CI 0.26–1.00). Furthermore, there was substantial evidence that living alone was associated with a delay to EIS (adj. HR = 0.63; 95% CI 0.43–0.92). These results were held after Bonferroni corrections (Table 2).

**Table 2:** Sociodemographic factors associated with delay to EIS (*n* = 318) analysed using Cox multiple regression

Characteristics	Median delay to EIS (IQR) days	Unadjusted hazard ratio (HR)	95% CI	Adjusted hazard ratio (HR)	95% CI	Bonferroni corrected 95% CI
Age-Band						
18–25	62 (9–1483)	1.00		1.00		
26–35	282 (22–1697)	0.66	0.50–0.87***	0.70	0.52–0.94**	n/a
Gender						
Male	64 (9–1508)	1.00				
Female	400 (24–1703)	0.65	0.51–0.89***	0.60	0.44–0.80***	n/a
Ethnicity						
White British	67 (14–1694)	1.00		1.00		
Black African	43 (9–1484)	1.14	0.78–1.67	1.17	0.79–1.71	− 0.47 to 0.71
Black Caribbean	118 (10–1583.5)	0.94	0.59–1.48	1.08	0.68–1.72	− 0.55 to 0.85
White other (non-British)	181 (22–1716)	0.87	0.53–1.42	0.98	0.59–1.61	− 0.87 to 0.72
Asian	1389 (13–1711)	0.63	0.34 – 1.16	0.72	0.38–1.35	− 1.61 to 0.44
Mixed	740 (13—1617)	0.79	0.40 – 1.57	0.79	0.40–1.58	− 1.21 to 0.84
Other	1336 (55–1869)	0.56	0.24 – 1.09	0.51	0.26–1.00**	− 1.86 to 0.38
Relationship status						
Single	120 (14–1633)	1.10	0.78–1.56	0.92	0.63–1.35	− 0.38 to 0.53
Married / Steady relationship	103 (12–1709.5)	1.00		1.00		
Divorced/Separated	79 (10–1891)	1.25	0.71–2.21	1.55	0.89–2.72	− 0.37 to 1.07
Employment						
Unemployed	282 (18—1703)	0.79	0.54–1.16	0.81	0.55–1.20	− 027 to 0.67
Student	64.5 (9—1459)	1.29	0.83–2.01	1.15	0.70–1.89	− 0.74–0.45
Employed	121 (15—1587)	1.00		1.00		
Lives with						
Alone	1336 (29—1715)	0.67	0.47–0.96***	0.63	0.43–0.92***	0.00–0.90
Family/relatives	79 (11—1609)	1.00		1.00		
Other	108 (13—1508)	0.74	0.47–1.13	0.80	0.50–1.29	− 0.79 to 0.36

*IQR* interquartile range, *CI* confidence interval

*P ≤ 0.1; **p ≤ 0.05 ***p ≤ 0.01

Model adjusted for age, gender, ethnicity, living circumstances and duration of untreated psychosis

Delay to EIS by clinical and pathways to care characteristics.

Table 3 shows the breakdown of delay to EIS according to pathways to care and clinical characteristics. There were significant differences in the delay to EIS by pathways to care and help-seeking characteristics. We found that family involvement in help-seeking was strongly associated with a shorter delay to EIS (adj. HR = 1.37; 95% CI 1.01–1.85). Conversely, a prolonged delay to EIS was associated with previous psychiatric service use (i.e., before the onset of psychosis) (adj. HR = 0.40; 95% CI 0.26–0.61). As seen above, the results in these models were held after Bonferroni adjustments (Table 3).

**Table 3:** Clinical and pathways to care factors associated with delay to EI (*n* = 318) analysed using Cox multiple regression

Characteristics	Median delay to EIS (IQR) days	Unadjusted hazard ratio (HR)	95% CI	Adjusted hazard ratio (HR)	95% CI	Bonferroni corrected 95% CI
Time of FEP contact						
Office hours	96 (12–1659)	1.00		1.00		
Out of hours	103 (16.5–1587.5)	0.99	0.74–1.32	0.89	0.66–1.21	n/a
Family involvement						
No	196.5 (21–1668.5)	1.00		1.00		
Yes	52.5 (9–1498)	1.47	1.11–1.94***	1.37	1.01–1.85**	n/a
Source of referral						
GP	77 (14–1583)	1.00		1.00		
A&E	103 (13–1593)	0.91	0.66–1.26	0.92	0.92–1.28	− 0.52–0.36
Police/Criminal Justice system	308.5 (18–1650.5)	0.84	0.523–1.31	0.96	0.96–1.54	− 0.67 to 0.59
Other	198 (9–1891)	0.64	0.38–1.07	0.70	0.70–1.17	− 1.03 to 0.33
Duration of untreated psychosis						
Short (≤ 6 months)	121 (11—1670)	1.00		1.00		
Long (> 6 months)	87 (20—1576)	1.18	0.89–1.57	1.25	0.93–1.68	n/a
Mode of onset						
Acute	81 (10–1609)	1.00		1.00		
Moderate	1399 (22.5–1716.5)	0.66	0.442–1.04	0.73	0.46–1.17	− 0.92 to 0.31
Gradual	73 (15–1633)	0.92	0.61–1.39	0.98	0.59 to 1.35	− 0.66 to 0.44
Insidious	87 (14–1587)	0.935	0.67–1.36	0.99	0.66–1.50	− 0.55 to 0.55
Mode of contact						
Community	89 (13–1532)	1.00		1.00		
Inpatient	108.3 (15–1696)	0.86	0.65–1.15	0.89	0.66–1.19	n/a
Involuntary admission at FEP						
No	101 (18.5–1633.5)	1.00		1.00		
Yes	88.5 (10–1648)	1.04	0.75–1.43	1.10	0.79–1.55	n/a
Previous psychiatric service use						
No	68.5 (10 – 1513.5)	1.00		1.00		
Yes	1614 (55–1892)	0.482	0.328–0.63***	0.40	0.26–0.61***	n/a

IQR, interquartile range. CI, confidence interval

*P ≤ 0.1; **p ≤ 0.05 ***p ≤ 0.01

Model adjusted for age, gender, ethnicity, living circumstances and duration of untreated psychosis

### Length of stay with EI

The overall median length of stay with EIS was 130 (IQR 0–663) days. Table 4 shows the length of stay with EIS by sociodemographic, clinical, and pathways to care characteristics. We found no evidence of differences in length of stay with EIS by any of our independent variables.

**Table 4:** Sociodemographic, clinical and pathways to care characteristics associated with length of EI stay (*n* = 318) analysed using negative binomial regression

Characteristics	Median length of stay (IQR) days	Unadjusted IRR	95% CI	Adjusted IRR	95% CI	Bonferroni corrected 95% CI
Age-Band						
18–25	213.5 (0- 711)	1.00		1.00		
26–35	36 (0–609)	0.81	0.46–1.42	0.80	0.44–1.43	n/a
Gender						
Male	221 (0 – 729.5)	1.00		1.00		
Female	32 (0–556)	0.79	0.45–1.39	0.75	0.41–1.36	n/a
Ethnicity						
White British	97 (0–686)	1.00		1.00		
Black African	253 (0–609)	1.08	0.49–2.35	1.10	0.49–2.48	− 1.17 to 1.37
Black Caribbean	373 (0–872.5)	1.29	0.51–3.25	1.35	0.52–3.45	− 1.24 to 1.75
White other (non- British)	207.5 (0–771)	1.07	0.40–2.91	1.08	0.40–2.91	− 1.51 to 1.70
Asian	0 (0–276)	0.63	0.20–1.94	0.73	0.22–2.36	− 2.48 to 1.27
Mixed	56.5 (0–663)	1.03	0.28–3.81	1.06	0.28–3.98	− 1.85 to 2.32
Other	0 (0–232)	0.57	0.28–1.86	0.44	0.13–1.52	− 2.74 to 1.14
Relationship status						
Single	123 (0–655)	0.28	− 0.64 to 0.70	1.12	0.53–2.32	− 1.17 to 0.71
Married / Steady relationship	130 (0–684)	1.00		1.00		
Divorced/Separated	219 (0–1092)	0.49	− 0.69 to 1.68	1.71	0.43–6.7	− 1.12 to 2.20
Employment						
Unemployed	63.5 (0–663)	0.85	0.40–1.80	0.79	0.36–1.72	− 0.99 to 1.18
Student	141 (0–586)	0.90	0.35–2.33	0.84	0.29–2.45	− 1.15 to 1.44
Employed	290 (0–636)	1.00		1.00		
Lives with						
Alone	0 (0–589)	0.79	0.39–1.56	0.77	0.37–1.60	n/a
Family/relatives	209 (0–711)	1.00		1.00		
Other	4147.5 (0–562)	0.74	0.30–1.56	0.72	0.29–1.72	n/a
Time of FEP contact						
Office hours	125.5 (0–657)	1.00				
Out of hours	182.5 (0–707.5)	1.11	0.62–1.97	0.94	0.51–1.77	n/a
Family involvement						
No	62.5 (0–574)	1.00		1.00		
Yes	300 (0–772)	1.39	0.79–2.47	1.22	0.65–2.30	n/a
Source of referral						
GP	199 (0–711)	1.00		1.00		
A&E	152 (0–663)	0.98	0.50–1.88	0.85	0.43–1.67	− 1.19 to 0.73
Police / Criminal Justice system	80.5 (0–782.5)	1.12	0.45–2.75	1.17	0.44–3.14	− 1.47 to 1.20
Other	90 (0–534)	0.73	0.28–1.87	0.73	0.26–1.91	− 1.94 to 0.82
Duration of untreated psychosis						
Short (≤ 6 months)	126 (0–636)	1.00		1.00		
Long (> 6 months)	161 (0–732)	1.13	0.63–2.02	1.20	0.59–2.45	n/a
Mode of onset						
Acute	240.5 (0–636)	1.00		1.00		
Moderate	0 (0–368)	0.77	0.32–1.85	0.73	0.30–1.78	− 1.44 to 1.11
Gradual	249 (0–771)	1.14	0.50–2.59	1.20	0.52–2.79	− 0.94 to 1.48
Insidious	129 (0–726)	1.04	0.50–2.15	1.03	0.46–2.30	− 0.84 to 1.33
Mode of contact						
Community	187.5 (0–726.5)			1.00		
Inpatient	101 (0–594)	0.88	0.50–1.55	0.81	0.45–1.47	n/a
Involuntary admission at FEP						
No	130 (0–707.5)	1.00		1.00		
Yes	133 (0–594)	1.04	0.55–1.97	1.00	0.50–1.98	n/a
Previous psychiatric service use						
No	210.5 (0–740)	1.00				
Yes	0 (0–534)	0.70	0.35–1.39	0.66	0.32–1.35	n/a

*IQR* interquartile range, *CI* confidence interval

*P ≤ 0.1; **p ≤ 0.05 ***p ≤ 0.01

Model adjusted for age, gender, ethnicity, living circumstances and duration of untreated psychosis

## Discussion

### Main findings

Our results suggest there are key sociodemographic and pathways to care indicators that influence time to early intervention service, both as protective and risk factors. There was evidence that sociodemographic factors, including female gender, older age, ethnicity, and living alone, were strongly associated with longer delays in accessing an EIS. Regarding pathways to care and clinical characteristics, our data showed that patients who had family involvement in their help-seeking were able to access EIS quicker than those without family involvement. Conversely, previous mental health service use before the onset of psychosis was strongly associated with prolonged delay to EIS. There was no strong evidence of sociodemographic and pathways to care differences in length of stay with EIS.

### Methodological considerations

Our study has key methodological strengths, including a large cohort of people with first episode psychosis, which enabled us to control for various confounding factors. This study adds to previous work in several ways. First, in contrast to some earlier studies, we followed up our cohort for 6 years after the first presentation for psychosis, leveraging a reliable estimate of the length of time to reach an EIS. Second, we used Cox proportional hazard and negative binomial models appropriate for our two outcomes (time to ESI and length of stay with EIS) rather than employing a non-parametric linear regression model, e.g., using log transformation, which would be less sensitive to outliers. Third, our sample is representative of the catchment area population of patients seen by an inner city mental health service.

Despite these strengths, our findings need to be interpreted with some limitations in mind. The cross-sectional nature of our case identification at the first presentation for psychosis meant that we were unable to capture the length of the help-seeking period outside secondary mental health services; therefore, our estimate of delay to EIS may be biased. While we adjusted for sociodemographic and pathways to care factors, our results could still be confounded by unmeasured characteristics of the patient that were more likely to have a shorter delay to EIS or likely to stay longer with EIS. For example, we did not measure the reasons for discharge, discontinuation of treatment or disengagement with EIS, which may have provided some insights into possible relationships between length of stay and patients’ characteristics. Later, we discuss the possible influence of the Access and Waiting Time Standards and how our findings compare to other studies. Whilst we used complete data (*n* = 318) in our multivariable analysis, our results may still be biased due to the missing data on 25 patients.

### Interpretations of findings and relationship to previous studies

#### Factors associated with delay to EIS

Our findings are consistent with previous evidence [5, 15, 32]. Several previous studies have highlighted the significance of family involvement in help-seeking for psychosis [18, 33, 34]. The work presented here extends our understanding of the role of the family in successfully reaching EIS not only during the first presentation for psychosis but also in receiving treatment in the appropriate specialist service. This is further illustrated in the Canadian Prevention and Early Intervention for Psychosis Program (PEPP), with primary objective of reducing delay to EIS, whereby anyone can refer a patient without the bureaucracy of navigating other primary or secondary care services [35]. The PEPP study authors found that 60% of the referrals were made by or involved family members [35]. We observed a range of factors associated with longer delay to EIS, such as being older, living alone, being a member of an ethnic minority group, and having previous mental health service use. These issues have been reported in previous research [36-38]. Birchwood and colleagues (2013), in their study of 348 FEP patients, showed that the greatest contribution to delay to EIS came from delays within mental health services, followed by help-seeking delays [15].

Further, in a recent qualitative study of pathways to EIS among FEP and at-risk mental state of psychosis patients, Allan et al. (under review) show that many of the eleven participants they interviewed had complex pathways to care; the majority had negative experiences, stating not being listened to or unheard, and having multiple contacts with different services before reaching EIS [39]. In our sample, we found that mental health service delays experienced by patients who may have presented with other psychiatric disorders before the manifestation of psychosis contributed to the prolonged period of reaching EIS. In contrast to some previous studies, we did not find strong associations between the duration of untreated psychosis and delay to EIS [15]. This could be due to the differences in the definition of DUP. For example, Birchwood et al. (2013) defined DUP as the time between the onset of positive symptoms of psychosis and the date of the first antipsychotic treatment [15]. However, we recognise that pathways to care: the time between onset, help-seeking, and receiving appropriate treatment is complex [40], and people with FEP often experience substantial delays and multiple help-seeking contacts before starting treatment [40, 41]. Our study provides insights into delays to EIS after the initial contact with mental health services when presenting with symptoms of psychosis.

The influence of gender, culture and illness belief of psychosis on delay to EIS is also noteworthy. Our findings show that belonging to an ‘other’ ethnic group (consisting of people from the Middle East, South America and any other ethnic group) predicted a longer delay to EIS. This is important, because strong evidence of the association between ethnicity and EI delay was revealed after adjusting for confounders (i.e., age, gender, living circumstances and DUP). To make sense of this finding, it is worth considering the role of gender in the manifestation of psychosis and help-seeking behaviour. It is widely documented that the rate of psychosis is higher among older women than older men [42, 43]. We showed in our previous reports from the CRIS-FEP sample that women were less likely to access EIS service compared with men (37% vs 63%, respectively) [44] and that they were more likely to be members of ethnic minority groups [17]. Gender plays a significant role in identity; as such, different cultures perceive gender roles and expectations differently [45]. For example, in some cultures or societies (e.g., South America, the Middle East, and Asia), people may believe that mental illness could be caused either by spirits or supernatural powers [45-48]; hence such beliefs will inevitably influence help-seeking behaviour. Given the sizable diversity in our sample, e.g., age, gender, and ethnicity, it is reasonable to suggest that these factors may have influenced how the patients make sense of their distress, then try to understand the cause and what could help to alleviate the symptoms. The patient health belief along with their social context also demonstrate the loci of control e.g. internal or external, which in turn will affect the type of help and treatment sought [45]. For example, a patient from a non-western culture who has been exposed to trauma, discrimination, and racism may be mistrustful of others and reluctant to contact medical professionals for help, leading to a significant delay in receiving the appropriate treatment [49]. Stigma may also play a role in delays in reaching EIS. The links between stigma and help-seeking for psychosis have been established [50, 51]. In some studies, stigma has been shown to manifest itself in FEP and at-risk-mental-state patients as worries about being weak, different, or a failure [39]. Ultimately, these social determinants and fundamental variations in help-seeking behaviours influence health inequalities.

#### Length of stay with EIS and the associated factors

Despite the duration of care within EIS in our study catchment area being up to three years, our data show that patients had a median length of stay of 130.5 days. We did not find strong evidence of an association between sociodemographic, pathways to care characteristics and length of stay with EIS. However, as we have acknowledged in the limitations of this study, the lack of data on the reasons for discharge, discontinuation of treatment or disengagement with EIS may have limited the ability to detect the relationships between length of stay with EIS and these patient-level characteristics. Further research is warranted exploring such composite outcomes. Meanwhile, despite the widely documented evidence that early initiation of treatment in an EIS improves longer-term outcomes, the optimal stay with EIS has been a matter of ongoing debate. To date, only a handful of studies have been carried out to examine how long the ideal period of treatment is in an EIS. In the Danish large RCT study of OPUS II trial [52], which compared the effects of 5 years of EIS treatment for first episode schizophrenia spectrum disorder with the standard 2 years of EIS plus 3 years of treatment as usual, the authors showed that patients in the 5 years of OPUS treatment were more likely to remain in contact with specialist mental health services (90.4% v 55.6%, *P* < 0.001). However, they did not examine the role of sociodemographic or pathway to care characteristics in this finding. In another study from Hong Kong, the Early Assessment Service for Young People with Psychosis (EASY), Chang et al. (2015) investigated the effect of extending a specialised early intervention treatment for first-episode psychosis by one year. They found no significant between-group difference in discontinuation rate [22]. In both OPUS II and EASY studies, DUP was measured as the delay to EIS, so comparisons with our findings are made cautiously. However, it has been reported in many studies that if patients are treated in an EIS for 3 years and then transferred to a generic mental health service, the improvement in clinical and social outcomes may be lost [22, 53-55].

### Implications for clinical practice

A striking finding in this study was the role of previous mental health service use in the delay to EIS. Indeed, findings from the PEPP programme showed evidence of previous service use leading to greater delay in accessing an early intervention program [40, 55]. However, this important finding warrants further attention, particularly from a service provision perspective. It is possible that patients prefer to remain with the services they are familiar with, and the services are happy to provide continuity of care. Therefore, the patient stays in a non-EIS service rather than transfer to a new service where they do not know anyone. Another key challenge could lie in clinician bias and the thresholds and boundaries of the criteria used for assessing first episode psychosis because these vary across EI services. For some services, the threshold is quite strict, meaning patients meet the criteria for severe illness, e.g., schizophrenia, in terms of symptoms and duration [19]. For others, a one-week duration of a frank psychotic symptom (usually based on positive symptoms—auditory hallucinations, disorganisation) is a sufficient threshold [56]. In addition, some of these services may or may not consider patients with other comorbidities [19]. It is, therefore, not surprising that patients with complex needs who have used mental health services previously may not be accepted for EIS due to the complexity of their needs. Fundamentally, psychosis can co-occur with other disorders, especially during the early stages of illness; and these comorbidities may be misattributed. For example, some individuals at the early stages of illness may present with symptoms of lesser severity and duration or non-psychotic symptoms such as anxiety and depression[57]. In addition, patients with pre-existing disorders, e.g., autism spectrum disorder presenting with FEP, are reported to be an under-identified population in EIS [58, 59]. The significance of help-seeking and intervening during the early phase of psychosis has been established [1, 33, 60]; and could potentially reduce DUP or prevent treatment delays [61]. Hence, there is a need for pragmatic screening procedures for accepting patients EIS, i.e., those that are sensitive to the biopsychosocial context of the early development of psychosis.

To reduce treatment delay for psychosis, policymakers and service commissioners need to ensure stronger links with local communities, whereby patients and families can access EIS quickly without having to navigate the prevailing layers of primary and secondary care systems. The Canadian PEEP program achieved a 72-h referral turnaround, because the formality of the referral process was removed. Therefore, patients, family members, schools, employers, and others concerned could refer someone to the service as needed. We acknowledge that this study was conducted before the introduction of new Access and Waiting Time Standards for early intervention for psychosis services in England, UK. However, evidence from the available research that has investigated the implementation of this policy suggests that meeting this 2-week target is heterogeneous. Some studies show that patients aged 35 years and above present to EIS with a complex need [62], but there is limited evidence on which factors influence pathways to care for patients over 35 years old. A recent study investigated the effect of the 14-day waiting time target for EIS after the first 6 months of its implementation [63]. It showed promising signs that patients in EIS had a higher chance of being seen and assessed within the waiting time target. However, the authors chose the referral closest to the start of EIS treatment, which may have underestimated the waiting time if earlier referrals were relevant to the psychotic episode. In another service evaluation at the North-East London NHS Foundation Trust, Singh et al. (2018) set out to increase the speed at which referrals were processed through the early intervention service to meet the Access and Waiting Time Standard. Using multiple interventions, including improving staff awareness, changing the case allocation process, and improving the referral pathway, the proportion of patients seen and assessed within 2 weeks rose from 21 to 62% [64]. However, the referral sources were mainly from statutory organisations, e.g., mental health services, psychiatric liaison services, criminal justice/probation service, and primary care. Such referral sources are typical for many EI services across the country, meaning there is little or no opportunity for a self-referral or informal referral.

Over the last decade, partly for economic reasons, early intervention for psychosis services have become less age-restrictive, and their functions are increasingly evolving. At times, EI services are merged with standard mental health care services, making boundaries between services and fidelity to the original EI models [10, 15] diluted over time. With the ongoing financial constraints and increasing caseload of patients per EIS practitioner, there remains the risk that efforts to intervene in the prodrome phase, community awareness, and increased access to EIS will be affected. Also significant is the issue of workforce shortage; a recent British Medical Association report shows that since 2016, there has been a 21% increase in the number of people in contact with mental health services [65]. Recruitment into psychiatric specialities remains a key challenge, with many psychiatric specialities facing under-recruitment year after year. In recent times, the impact of the COVID-19 pandemic has put further strain on the overall health workforce. Consequently, staff shortages in mental health will affect EIS staff workload, well-being, and morale and impact their ability to provide good quality of care.

## Conclusions

Our analyses highlight the link between sociodemographic status, pathways to care, and delay to EIS, but also show the vulnerability faced by those with a previous mental health problem who later develop psychosis in receiving specialist treatment for psychosis. This research shows that the barriers to accessing early intervention services are beyond the time of initial referral but much later. Service-related factors play a crucial role in delays to EI services, as our data show that once patients are within the mental health system, they experience long waiting times. Some patients are not referred to specialist psychosis services at all. Initiatives that ameliorate indicators of social disadvantage are urgently needed to reduce health inequalities and improve clinical outcomes.

### Supplementary Information

Below is the link to the electronic supplementary material.

Supplementary file1 (DOCX 118 KB)

## Data Availability

No additional data are available.
