# Supplementary material for: Sociodemographic and clinical predictors of delay to and length of stay with early intervention for psychosis service: findings from the CRIS-FEP study
Source: Soc Psychiatry Psychiatr Epidemiol. 2023 Jun 23;59(1):25–36. doi: 10.1007/s00127-023-02522-z (PMC10799823; doi:10.1007/s00127-023-02522-z)
Supplement: Supplementary file 1 — Supplementary file1 (DOCX 118 KB) [file 127_2023_2522_MOESM1_ESM.docx]

Title: Sociodemographic and clinical predictors of delay to and length of stay with early intervention for psychosis service: findings from the CRIS-FEP study

**Authors:**

Sherifat Oduola ^1, 3^, Tom K. J. Craig^2,3^ , Eduardo Iacoponi^3^ , Alastair Macdonald ^2,3^ and Craig Morgan^2,3^

**Affiliations**

1. School of Health Sciences, University of East Anglia, Norwich Research Park, Norwich NR4 7TJ, UK
2. Department of Health Service and Population Research, Institute of Psychiatry, Psychology & Neuroscience, King’s College London, De Crespigny Park, Denmark Hill, London SE5 8AF, UK
3. South London & Maudsley NHS Foundation Trust, Denmark Hill, London SE5 8AZ, UK

**Correspondence to:**

Sherifat Oduola, School of Health Sciences, University of East Anglia. Tel: 01603597024. E-mail: [s.oduola@uea.ac.uk](mailto:s.oduola@uea.ac.uk)

Abstract Word Count: 241

Text Body Word Count: 4,446

Tables: 4

Figures: 1

FUNDING

This work was supported by UK Medical Research Council (Ref: G0500817), the Wellcome Trust (Grant Number: WT087417), the European Union (European Community’s Seventh Framework Program (grant agreement No. HEALTH-F2-2009-241909) (Project EU-GEI)). The views expressed are those of the authors and not necessarily those of the funding bodies.

ACKNOWLEDGEMENTS

This paper represents independent research supported by the National Institute for Health Research (NIHR) Biomedical Research Centre at South London and Maudsley NHS Foundation Trust and King’s College London. The views expressed are those of the authors and not necessarily those of the NHS, the NIHR or the Department of Health and Social Care.

# Abstract

**Purpose:** We investigated the influence of sociodemographic and clinical characteristics on delay to early intervention service (EIS) and the length of stay (LOS) with EIS.

**Methods:** We used incidence data linked to the Clinical Record Interactive Search - First Episode Psychosis (CRIS-FEP) study. We followed the patients from May 2010 to March 2016. We performed multivariable Cox regression to estimate hazard ratios of delay to EIS. Negative binomial regression was used to determine LOS with EIS by sociodemographic and clinical characteristics, controlling for confounders.

**Results:** 343 patients were eligible for an EIS, 34.1% of whom did not receive the service. Overall, the median delay to EIS was 120 days (IQR; 15-1668); and the median LOS was 130.5 days (IQR: 0 – 663). We found that women (adj.HR: 0.58; 95%CI: 0.42-0.78), living alone (adj.HR: 0.63; 95%CI: 0.43 – 0.92) and ethnicity (‘Other’: adj.HR: 0.47; 95%CI: 0.23-0.98) were associated with prolonged delay to EIS. However, family involvement in help-seeking for psychosis (adj.HR:1.37; 95%CI:1.01 – 1.85) was strongly associated with a shorter delay to EIS. Patients who have used mental health services previously also experienced long delays to EIS.

**Conclusions:** Our analyses highlight the link between sociodemographic status, help-seeking behaviours, and delay to EIS. Our findings also show the vulnerability faced by those with a previous mental health problem who later develop psychosis in receiving specialist treatment for psychosis. Initiatives that ameliorate indicators of social disadvantage are urgently needed to reduce health inequalities and improve clinical outcomes.

Keywords: early intervention psychosis, treatment delays, first episode psychosis, pathways to care, length of stay.

# Introduction

First episode psychosis often begins with a prodrome phase of a low threshold of symptoms and altered functioning before the onset of frank psychosis. The onset of psychosis typically occurs when people are young, and they may be reluctant to seek help because of the blur between psychotic symptoms and normal developmental changes [1, 2]. Timely access to specialist early intervention for psychosis has been shown to halt poorer outcomes [3-5]. Therefore, improving the short and long-term outcomes of psychosis has been the preoccupation of service providers globally [5, 6]. Since the late 1990s and 2000s, early intervention services for psychosis have been established in many high-and middle-income countries[7-9]. Several of these have been carefully evaluated and showed that early intervention for psychosis care is superior in improving clinical and functional outcomes compared with standard care [10-14]. Some studies have highlighted individual, clinical, and service-related factors impeding access to EI service [15, 16]. For example, living alone, unemployment and social isolation are linked to a longer duration of untreated psychosis [16]. In terms of pathways to care, ethnicity is well documented as a risk for involuntary admissions [17] and criminal justice system involvement [18].

To establish parity of esteem between physical and mental health, the UK government introduced the Access and Waiting Time Standard for early intervention for psychosis services in England [19]. It recommended that adults presenting with a first episode of psychosis (FEP) should start treatment in early intervention for psychosis services within two weeks of referral [20]. However, despite the widely documented evidence that early initiation of treatment in early intervention for psychosis service improves longer-term outcomes, the optimal duration of stay with EI service has been a matter of ongoing debate. Only a handful of studies have been conducted to investigate the ideal period of stay and treatment with an EIS [21, 22].

To date, much of our understanding of the factors associated with delay to an early intervention service (EIS) has been gleaned through the lens of the duration of untreated psychosis [16, 23, 24], which considers the time between the onset of symptoms and first contact with a mental health service or first antipsychotic treatment; therefore our understanding specifically in delay in reaching an EIS is distorted. Hence, high-quality research on the influence of sociodemographic, clinical, and pathways to care characteristics on delay to reaching an EIS and, subsequently how long patients stay with an EIS is limited. A better understanding of factors associated with delay in reaching an early intervention for psychosis service will inform the development of strategies to ameliorate them. From the few available studies that focus on pathways to EIS, several are based on cross-sectional samples and do not account for the non-randomness of the length of the pathway to EIS [5, 15]. To our knowledge, there has not been a longitudinal cohort study that investigated the associations between sociodemographic clinical characteristics, length of delay to EIS, and length of stay with EIS. To address these gaps, in this study, we use an epidemiologically derived cohort of first episode psychosis patients. We sought to (a) estimate the length of delay to an EIS from first contact for psychosis, (b) examine sociodemographic, pathways to care, and clinical factors associated with delay to EIS, and (c) determine the length of stay with EIS and the associated factors.

# Methods

## Samples

The study was conducted in two inner city areas of London, served by the South London and Maudsley NHS Foundation Trust (SLaM). These are the London boroughs of Lambeth and Southwark, with a combined population of 625,300 people [25]

## Inclusion/exclusion criteria

The present study is part of a larger incidence study conducted between May 2010 and April 2012 [26]. We included participants if they were residents in the London boroughs of Lambeth or Southwark, (b) aged 18-64 years old (inclusive) at presentation, (c) with a clinical diagnosis of a psychotic disorder (i.e., ICD F20-29, F30-33), and (d) were in first contact with mental health services for psychosis. Exclusion criteria were: (a) evidence of psychotic symptoms with an organic cause, (b) transient psychotic symptoms resulting from acute intoxication, and (c) previous contact with services for psychotic symptoms.

At the time of this study, early intervention for psychosis services at SLaM typically offered a 3-year duration of treatment and support. The age eligibility criterion for accessing an EIS in SLaM was 18 – 35 years; this was before the introduction of the Access and Waiting Time Standard, i.e., 1 April 2016, when the upper age limit was extended to 65 year. Therefore, we restricted our analyses to those that met the earlier age (i.e. 18-35 years) criterion for an EIS.

## Study design, setting, and participants

The participants included in this study were drawn from an incidence cohort of patients with first episode psychosis (i.e., ICD F20-29, F30-33) assembled for the Clinical Record Interactive Search—First Episode Psychosis (CRIS-FEP) study[26]. In brief, we identified all patients presenting to the South London and Maudsley NHS Trust adult mental health services in Lambeth and Southwark for the first time with a psychotic disorder between May 2010 and April 2012. We used the South London and Maudsley NHS Trust (SLaM) Clinical Records Interactive Search (CRIS) system [27], which provides fully de-identified access to all SLaM electronic clinical records.

## Outcome variables and covariates

The primary outcomes were:

1. Time to acceptance by an early intervention service, measured from the date of the first presentation for psychosis or the discharge date from inpatient admission for psychosis (if admitted at first presentation). This time is considered to represent the beginning of delay to an EIS following a presentation for first episode psychosis in SLaM. Patients were followed until the date of acceptance to an EIS, end of the study (31 March 2016), or date of discharge from SLaM services, whichever came first.
2. Length of stay with EIS, measured from the date of acceptance to an EI service, and patients were followed until the end of the study (31 March 2016) or date of discharge from EI services or death, whichever came first. We considered this time to represent the start of the duration of time individuals received treatment from an EIS.

## Covariates

Sociodemographic, clinical, and pathways to care characteristics were collected as covariates: age, gender, ethnicity, living circumstances, employment status, duration of untreated psychosis, mode of onset of psychosis, and source of referral. Data on demographic and social circumstances were extracted from the patient’s de-identified electronic clinical records guided by the Medical Research Council Sociodemographic schedule MRC-SDS [28]. Ethnicity was self-ascribed and recorded in clinical records. We categorised ethnicity according to the 18 categories of the 2011 UK Census [29]. For the purpose of analysis, we collapsed the ethnic groups into seven categories in line with our previous studies [16, 26] as follows: white British, black Caribbean (black Caribbean and other black), black African, Asian (Indian, Pakistani, Bangladeshi, Chinese), white non-British (white Irish, white Gypsy, white Other), other (Arab, any other ethnic group) and mixed (all mixed groups).

Data relating to pathways to care, duration of untreated psychosis, and EIS encounters were also extracted from the patient’s de-identified electronic clinical records using the Personal and Psychiatric History Schedule (PPHS) [30]. Duration of untreated psychosis was measured from the date of onset of psychotic symptoms to the date of first contact with SLaM for psychosis [16].

## Statistical analysis

Stata (version 15) software was used to analyse the data [31]. Numbers, frequencies, mean, and medians, along with the standard deviation and interquartile range, were used as appropriate to describe the sample. Descriptive statistics for dependent and independent variables were obtained as median with interquartile range, with the two outcomes of delay to EIS and length of stay with EIS. Kaplan-Meier survival analysis and multivariable Cox regression were used to assess associations between delay to EIS and covariates. First, we performed univariable Cox regression for estimates of unadjusted hazard ratios for the delay to EIS, then adjusted for a-priori confounders (i.e., age, gender, ethnicity, living circumstances, and duration of untreated psychosis). The hazard ratios derived from Cox regression analyses represent the probability of receiving an EIS during the follow-up period. Therefore, a hazard ratio greater than 1 denotes an association of an independent variable with the shortest time to EIS.

To assess the association of independent variables with the length of stay (LOS) with EIS, we employed negative binominal regression whilst taking into account the follow-up period using the *exposure* option in Stata for unadjusted and adjusted incidence rate ratios of LOS. Negative binomial regression models were used to overcome the over-dispersion of zero, and the data were not normally distributed (Pearson goodness-of-fit X^2^ =1991.0, df = 342, p<0.0001).

We addressed missing data in our multivariable regression analyses by including only patients with complete data on all variables included in the models. We conducted Bonferroni adjustments for multiple comparisons when relevant.

## Ethical approval

The CRIS system was approved as an anonymised dataset for secondary analysis by the Oxfordshire Research Ethics Committee (reference 08/H0606/71). We obtained local approval for this study via the CRIS Oversight Committee at the BRC South London and Maudsley NHS Foundation Trust (reference: 09-041).

# Results

Three hundred and forty-three patients aged 18-35 years were eligible for an EIS. The mean age was 26.1 (sd, 5.0) years, there were more men (n, 198(57.7%)) than women, and black African patients (n, 95 (27.7%)) were the largest proportions of the sample. The median duration of untreated psychosis was 87 (IQR: 14- 410) days; 142 (41.4%) patients were referred to mental health services via the Accident and Emergency department. Table 1 describes the study sample characteristics.

Insert Table 1

## Delay to EIS by sociodemographic characteristics

During the follow-up period, 318 patients with complete data constituted 701.9 person-years at risk, of whom 222 received EIS, meaning 34.9% of the eligible patients did not receive an EIS. The median delay to EIS was 120 (IQR; 15-1668) days. Kaplan-Meier plot (Figure 1) shows the distribution of delay to EIS overtime. Table 2 presents the relationships between delay to EIS and sociodemographic characteristics. We found strong evidence of delay to EIS in older patients (adj.HR= 0.70; 95%CI=0.52-0.94), among women (adj. HR=0.60; 95%CI: 0.44-0.80) and patients of ‘other’ ethnic groups (adj. HR=0.51; 95%CI:0.26 -1.00). Furthermore, there was substantial evidence that living alone was associated with a delay to EIS (adj. HR=0.63; 95%CI: 0.43 – 0.92). These results were held after Bonferroni corrections (Table 2).

## Delay to EIS by clinical and pathways to care characteristics

Table 3 shows the breakdown of delay to EIS according to pathways to care and clinical characteristics. There were significant differences in the delay to EIS by pathways to care and help-seeking characteristics. We found that family involvement in help-seeking was strongly associated with a shorter delay to EIS (adj. HR=1.37; 95%CI:1.01 – 1.85). Conversely, a prolonged delay to EIS was associated with previous psychiatric service use (i.e., before the onset of psychosis) (adj. HR=0.40; 95%CI:0.26 – 0.61). As seen above, the results in these models were held after Bonferroni adjustments (Table 3).

Insert Figure 1

Insert Table 2

Insert Table 3

## Length of stay with EI

The overall median length of stay with EIS was 130 (IQR: 0 – 663) days. Table 4 shows the length of stay with EIS by sociodemographic, clinical, and pathways to care characteristics. We found no evidence of differences in length of stay with EIS by any of our independent variables.

Insert Table 4

# Discussion

## Main findings

Our results suggest there are key sociodemographic and pathways to care indicators that influence time to early intervention service, both as protective and risk factors. There was evidence that sociodemographic factors, including female gender, older age, ethnicity, and living alone, were strongly associated with longer delays in accessing an EIS. Regarding pathways to care and clinical characteristics, our data showed that patients who had family involvement in their help-seeking were able to access EIS quicker than those without family involvement. Conversely, previous mental health service use before the onset of psychosis was strongly associated with prolonged delay to EIS. There was no strong evidence of sociodemographic and pathways to care differences in length of stay with EIS.

## Methodological considerations

Our study has key methodological strengths, including a large cohort of people with first episode psychosis, which enabled us to control for various confounding factors. This study adds to previous work in several ways. First, in contrast to some earlier studies, we followed up our cohort for six years after the first presentation for psychosis, leveraging a reliable estimate of the length of time to reach an EIS. Second, we used Cox proportional hazard and negative binomial models appropriate for our two outcomes (time to ESI and length of stay with EIS) rather than employing a non-parametric linear regression model, e.g., using log transformation, which would be less sensitive to outliers. Third, our sample is representative of the catchment area population of patients seen by an inner-city mental health service.

Despite these strengths, our findings need to be interpreted with some limitations in mind. The cross-sectional nature of our case identification at the first presentation for psychosis meant that we were unable to capture the length of the help-seeking period outside secondary mental health services; therefore, our estimate of delay to EIS may be biased. While we adjusted for sociodemographic and pathways to care factors, our results could still be confounded by unmeasured characteristics of the patient that were more likely to have a shorter delay to EIS or likely to stay longer with EIS. For example, we did not measure the reasons for discharge, discontinuation of treatment or disengagement with EIS, which may have provided some insights into possible relationships between length of stay and patients’ characteristics. Later, we discuss the possible influence of the Access and Waiting Time Standards and how our findings compare to other studies. Whilst we used complete data (n=318) in our multivariable analysis, our results may still be biased due to the missing data on twenty-five patients.

## Interpretations of findings and relationship to previous studies

***Factors associated with delay to EIS***

Our findings are consistent with previous evidence [5, 15, 32]. Several previous studies have highlighted the significance of family involvement in help-seeking for psychosis [18, 33 , 34]. The work presented here extends our understanding of the role of the family in successfully reaching EIS not only during the first presentation for psychosis but also in receiving treatment in the appropriate specialist service. This is further illustrated in the Canadian Prevention and Early Intervention for Psychosis Program (PEPP), with primary objective of reducing delay to EIS, whereby anyone can refer a patient without the bureaucracy of navigating other primary or secondary care services [35]. The PEPP study authors found that 60% of the referrals were made by or involved family members [35]. We observed a range of factors associated with longer delay to EIS, such as being older, living alone, being a member of an ethnic minority group, and having previous mental health service use. These issues have been reported in previous research [36-38]. Birchwood and colleagues (2013), in their study of 348 FEP patients, showed that the greatest contribution to delay to EIS came from delays within mental health services, followed by help-seeking delays [15].

Further, in a recent qualitative study of pathways to early intervention service (EIS) among FEP and at-risk mental state of psychosis patients, Allan et al. (under review) show that many of the eleven participants they interviewed had complex pathways to care; the majority had negative experiences, stating not being listened to or unheard, and having multiple contacts with different services before reaching EIS [39]. In our sample, we found that mental health service delays experienced by patients who may have presented with other psychiatric disorders before the manifestation of psychosis contributed to the prolonged period of reaching EIS. In contrast to some previous studies, we did not find strong associations between the duration of untreated psychosis and delay to EIS [15]. This could be due to the differences in the definition of DUP. For example, Birchwood et al. (2013) defined DUP as the time between the onset of positive symptoms of psychosis and the date of the first antipsychotic treatment [15]. However, we recognise that pathways to care: the time between onset, help-seeking, and receiving appropriate treatment is complex [40], and people with FEP often experience substantial delays and multiple help-seeking contacts before starting treatment [40, 41]. Our study provides insights into delays to EIS after the initial contact with services when presenting with symptoms of psychosis.

The influence of gender, culture and illness belief of psychosis on delay to EIS is also noteworthy. Our findings show that belonging to an ‘other’ ethnic group (consisting of people from the Middle East, South America and any other ethnic group) predicted a longer delay to EIS. This is important because strong evidence of the association between ethnicity and EI delay was revealed after adjusting for confounders (i.e., age, gender, living circumstances and DUP). To make sense of this finding, it is worth considering the role of gender in the manifestation of psychosis and help-seeking behaviour. It is widely documented that the rate of psychosis is higher among older women than older men [42, 43]. We showed in our previous reports from the CRIS-FEP sample that women were less likely to access EIS service compared with men (37% vs 63% respectively) [44] and that they were more likely to be members of ethnic minority groups [17]. Gender plays a significant role in identity; as such, different cultures perceive gender roles and expectations differently [45]. For example, in some cultures or societies (e.g., South America, the Middle East, and Asia), people may believe that mental illness could be caused either by spirits or supernatural powers [45-48]; hence such beliefs will inevitably influence help-seeking behaviour. Given the sizable diversity in our sample, e.g., age, gender, and ethnicity, it is reasonable to suggest that these factors may have influenced how the patients make sense of their distress, then try to understand the cause and what could help to alleviate the symptoms. The patient health belief along with their social context also demonstrate the loci of control e.g. internal or external, which in turn will affect the type of help and treatment sought [45]. For example, a patient from a non-western culture who has been exposed to trauma, discrimination, and racism may be mistrustful of others and reluctant to contact medical professionals for help, leading to a significant delay in receiving the appropriate treatment [49]. Stigma may also play a role in delays in reaching EIS. The links between stigma and help-seeking for psychosis have been established [50, 51]. In some studies, stigma has been shown to manifest itself in FEP and at-risk-mental-state patients as worries about being weak, different, or a failure [39]. Ultimately, these social determinants and fundamental variations in help-seeking behaviours influence health inequalities.

***Length of stay with EIS and the associated factors***

Despite the duration of care within EIS in our study catchment area being up to three years, our data show that patients had a median length of stay of 130.5 days. We did not find strong evidence of an association between sociodemographic, pathways to care characteristics and length of stay with EIS. However, as we have acknowledged in the limitations of this study, the lack of data on the reasons for discharge, discontinuation of treatment or disengagement with EIS may have limited the ability to detect the relationships between length of stay with EIS and these patient-level characteristics. Further research is warranted exploring such composite outcomes. Meanwhile, despite the widely documented evidence that early initiation of treatment in an EIS improves longer-term outcomes, the optimal stay with EIS has been a matter of ongoing debate. To date, only a handful of studies have been carried out to examine how long the ideal period of treatment is in an EIS. In the Danish large RCT study of OPUS II trial [52], which compared the effects of five years of EIS treatment for first episode schizophrenia spectrum disorder with the standard two years of EIS plus three years of treatment as usual, the authors showed that patients in the five years of OPUS treatment were more likely to remain in contact with specialist mental health services (90.4% v 55.6%, P<0.001). However, they did not examine the role of sociodemographic or pathway to care characteristics in this finding. In another study from Hong Kong, the Early Assessment Service for Young People with Psychosis (EASY), Chang et al. (2015) investigated the effect of extending a specialised early intervention treatment for first-episode psychosis by one year. They found no significant between-group difference in discontinuation rate [22]. In both OPUS II and EASY studies, DUP was measured as the delay to EIS, so comparisons with our findings are made cautiously. However, it has been reported in many studies that if patients are treated in an EIS for three years and then transferred to a generic mental health service, the improvement in clinical and social outcomes may be lost [22, 53-55].

# Implications for clinical practice

A striking finding in this study was the role of previous mental health service use in the delay to EIS. Indeed, findings from the PEPP programme showed evidence of previous service use leading to greater delay in accessing an early intervention program [40, 55]. However, this important finding warrants further attention, particularly from a service provision perspective. It is possible that patients prefer to remain with the services they are familiar with, and the services are happy to provide continuity of care. So, the patient stays in a non-EIS service rather than transfer to a new service where they do not know anyone. A key challenge could lie in clinician bias and the thresholds and boundaries of the criteria used for assessing first episode psychosis because these vary across EI services. For some services, the threshold is quite strict, meaning patients meet the criteria for severe illness, e.g., schizophrenia, in terms of symptoms and duration [19]. For others, a one-week duration of a frank psychotic symptom (usually based on positive symptoms – auditory hallucinations, disorganisation) is a sufficient threshold [56]. In addition, some of these services may or may not consider patients with other co-morbidities [19]. It is, therefore, not surprising that patients with complex needs who have used mental health services previously may not be accepted for EIS due to the complexity of their needs. Fundamentally, psychosis can co-occur with other disorders, especially during the stages of illness; and these comorbidities may be misattributed. For example, some individuals at the early stages of illness may present with symptoms of lesser severity and duration or non-psychotic symptoms such as anxiety and depression[57]. In addition, patients with pre-existing disorders, e.g., autism spectrum disorder presenting with FEP, are reported to be an under-identified population in EIS [58, 59]. The significance of help-seeking and intervening during the early phase of psychosis has been established [1, 33, 60]; and could potentially reduce DUP or prevent treatment delays [61]. Hence, there is a need for pragmatic screening procedures for accepting patients EIS, i.e., those that are sensitive to the biopsychosocial context of the early development of psychosis.

To reduce treatment delay for psychosis, policymakers and service commissioners need to ensure stronger links with local communities, whereby patients and families can access EIS quickly without having to navigate the prevailing layers of primary and secondary care systems. The Canadian PEEP program achieved a 72-hour referral turnaround because the formality of the referral process was removed. So, patients, family members, schools, employers, and others concerned could refer to the service as needed. We acknowledge that this study was conducted before the introduction of new Access and Waiting Time Standards for early intervention for psychosis services in England, UK. However, evidence from the available research that has investigated the implementation of this policy suggests that meeting this two-week target is heterogeneous. Some studies show that patients aged 35 years and above present to EIS with a complex need [62], but there is limited evidence on which factors influence pathways to care for patients over 35 years old. A recent study investigated the effect of the 14-day waiting time target for EIS after the first six months of its implementation [63]. It showed promising signs that patients in EIS had a higher chance of being seen and assessed within the waiting time target. However, the authors chose the referral closest to the start of EIS treatment, which may have underestimated the waiting time if earlier referrals were relevant to the psychotic episode. In another service evaluation at the North-East London NHS Foundation Trust, Singh et al. (2018) set out to increase the speed at which referrals were processed through the early intervention service to meet the Access and Waiting Time Standard. Using multiple interventions, including improving staff awareness, changing the case allocation process, and improving the referral pathway, the proportion of patients seen and assessed within two weeks rose from 21% to 62% [64]. However, the referral sources were mainly from statutory organisations, e.g., mental health services, psychiatric liaison services, criminal justice/probation service, and primary care. Such referral sources are typical for many EI services across the country, meaning there is little or no opportunity for a self-referral or informal referral.

Over the last decade, partly for economic reasons, early intervention for psychosis services have become less age-restrictive, and their functions are increasingly evolving. At times, EI services are merged with standard mental health care services, making boundaries between services and fidelity to the original EI models [10, 15] diluted over time. With the ongoing financial constraints and increasing caseload of patients per EIS practitioner, there remains the risk that efforts to intervene in the prodrome phase, community awareness, and increased access to EIS will be affected. Also significant is the issue of workforce shortage; a recent British Medical Association report shows that since 2016, there has been a 21% increase in the number of people in contact with mental health services [65]. Recruitment into psychiatric specialities remains a key challenge, with many psychiatric specialities facing under-recruitment year after year. In recent times, the impact of the COVID-19 pandemic has put further strain on the overall health workforce. Consequently, staff shortages in mental health will only affect EIS staff workload, well-being, and morale and impact their ability to provide good quality of care.

## Conclusions

Our analyses highlight the link between sociodemographic status, pathways to care, and delay to EIS, but also show the vulnerability faced by those with a previous mental health problem who later develop psychosis in receiving specialist treatment for psychosis. This research shows that the barriers to accessing early intervention services are beyond the time of initial referral but much later. Service-related factors play a crucial role in delays to EI services, as our data shows that once patients are within the mental health system, they experience long waiting times. Some patients are not referred to specialist psychosis services at all. Initiatives that ameliorate indicators of social disadvantage are urgently needed to reduce health inequalities and improve clinical outcomes.

# References

1. Marwaha, S., et al., *Fifteen years on - early intervention for a new generation.* Br J Psychiatry, 2016. **209**(3): p. 186-8.

2. Murphy, B.P. and W.J. Brewer, *Early intervention in psychosis: strengths and limitations of services.* Advances in Psychiatric Treatment, 2018. **17**(6): p. 401-407.

3. Drake, R.J., et al., *Causes and consequences of duration of untreated psychosis in schizophrenia.* Br J Psychiatry, 2000. **177**: p. 511-5.

4. Bird, V., et al., *Early intervention services, cognitive–behavioural therapy and family intervention in early psychosis: systematic review.* The British Journal of Psychiatry, 2010. **197**(5): p. 350-356.

5. Bhui, K., S. Ullrich, and J.W. Coid, *Which pathways to psychiatric care lead to earlier treatment and a shorter duration of first-episode psychosis?* BMC Psychiatry, 2014. **14**: p. 72.

6. Marshall, M. and J. Rathbone, *Early Intervention for Psychosis.* Schizophrenia Bulletin, 2011. **37**(6): p. 1111-1114.

7. Nordentoft, M., *Specialised assertive intervention in early psychosis.* Lancet Psychiatry, 2015. **2**(1): p. 2-3.

8. Henry, L.P., et al., *The EPPIC follow-up study of first-episode psychosis: longer-term clinical and functional outcome 7 years after index admission.* The Journal of clinical psychiatry, 2010. **71**(6): p. 716.

9. Chen, E.Y.H., et al., *Early Intervention for Psychosis Intervention: A Real-Life System in Hong Kong.* Schizophrenia Bulletin, 2015. **41**: p. S165-S165.

10. Craig, T.K., et al., *The Lambeth Early Onset (LEO) Team: randomised controlled trial of the effectiveness of specialised care for early psychosis.* British Medical Journal, 2004. **329**(7474): p. 1067-69.

11. Birchwood, M., *Early intervention in psychotic relapse: Cognitive approaches to detection and management.* Behaviour Change, 1995.

12. Kuipers, E., et al., *An RCT of early intervention in psychosis: Croydon Outreach and Assertive Support Team (COAST).* Social psychiatry and psychiatric epidemiology, 2004. **39**(5): p. 358-363.

13. McGorry, P.D., et al., *Intervention in individuals at ultra-high risk for psychosis: a review and future directions.* Journal of Clinical Psychiatry, 2009. **70**(9): p. 1206.

14. Bertelsen, M., et al., *Five-year follow-up of a randomized multicenter trial of intensive early intervention vs standard treatment for patients with a first episode of psychotic illness: the OPUS trial.* Archives of general psychiatry, 2008. **65**(7): p. 762-771.

15. Birchwood, M., et al., *Reducing duration of untreated psychosis: care pathways to early intervention in psychosis services.* The British Journal of Psychiatry, 2013. **203**(1): p. 58-64.

16. Oduola, S., T.K.J. Craig, and C. Morgan, *Ethnic variations in duration of untreated psychosis: report from the CRIS-FEP study.* Soc Psychiatry Psychiatr Epidemiol, 2020.

17. Oduola, S., et al., *Compulsory admission at first presentation to services for psychosis: does ethnicity still matter? Findings from two population-based studies of first episode psychosis.* Social Psychiatry and Psychiatric Epidemiology, 2019. **54**(7): p. 871-881.

18. Morgan, C., et al., *Pathways to care and ethnicity. 2: Source of referral and help-seeking. Report from the AESOP study.* Br J Psychiatry, 2005. **186**: p. 290-6.

19. NHS England. *Implementing the Early Intervention in Psychosis Access and Waiting Time Standard: Guidance*. 2016 [cited 2023 Februrary 2023]; Available from: <https://www.nice.org.uk/guidance/qs80/resources/implementing-the-early-intervention-in-psychosis-access-and-waiting-time-standard-guidance-2487749725>.

20. NICE. *Psychosis and schizophrenia in adults*. 2015; Available from: <https://www.nice.org.uk/guidance/qs80>.

21. Albert, N., et al., *The effect of duration of untreated psychosis and treatment delay on the outcomes of prolonged early intervention in psychotic disorders.* NPJ Schizophr, 2017. **3**(1): p. 34.

22. Chang, W.C., et al., *Optimal duration of an early intervention programme for first-episode psychosis: randomised controlled trial.* Br J Psychiatry, 2015. **206**(6): p. 492-500.

23. Ehmann, T.S., et al., *Treatment delay and pathways to care in early psychosis.* Early Interv Psychiatry, 2014. **8**(3): p. 240-6.

24. Malla, A., et al., *A controlled evaluation of a targeted early case detection intervention for reducing delay in treatment of first episode psychosis.* Soc Psychiatry Psychiatr Epidemiol, 2014. **49**(11): p. 1711-8.

25. Impact on Urban Health. *Census 2021: How the population changed in our boroughs*. 2022 [cited 2022 October 2022]; Available from: <https://urbanhealth.org.uk/insights/data/census-2021-lambeth-southwark>.

26. Oduola, S., et al., *Change in incidence rates for psychosis in different ethnic groups in south London: findings from the Clinical Record Interactive Search-First Episode Psychosis (CRIS-FEP) study.* Psychol Med, 2021. **51**(2): p. 300-309.

27. Perera, G., et al., *Cohort profile of the South London and Maudsley NHS Foundation Trust Biomedical Research Centre (SLaM BRC) Case Register: current status and recent enhancement of an Electronic Mental Health Record-derived data resource.* BMJ open, 2016. **6**(3): p. e008721.

28. Mallett, R., *MRC Sociodeomgraphic Schedule*. 1997, Institute of Psychiatry, King's College London: Section of Social Psychiatry.

29. ONS. *Official Labour Market Statistics*. 2011 [cited 2022 2022]; Available from: <https://www.nomisweb.co.uk/>.

30. WHO, *Personal and Psychiatric History Schedule*, in *Geneva: World Health Organisation*. 1996.

31. StataCorp, *Stata Statistical Software: Release 15*, in *College Station, TX: StataCorp LLC.* 2017.

32. Thomas, S.P. and H.S. Nandhra, *Early intervention in psychosis: a retrospective analysis of clinical and social factors influencing duration of untreated psychosis.* Prim Care Companion J Clin Psychiatry, 2009. **11**(5): p. 212-4.

33. Allan, S.M., et al., *Pathways to care in at-risk mental states: A systematic review.* Early Interv Psychiatry, 2020.

34. Compton, M.T., et al., *Patient-level predictors and clinical correlates of duration of untreated psychosis among hospitalized first-episode patients.* J Clin Psychiatry, 2011. **72**(2): p. 225-32.

35. MacDonald, K., et al., *Description, evaluation and scale-up potential of a model for rapid access to early intervention for psychosis.* Early Interv Psychiatry, 2018. **12**(6): p. 1222-1228.

36. Archie, S., et al., *Ethnic diversity and pathways to care for a first episode of psychosis in Ontario.* Schizophr Bull, 2010. **36**(4): p. 688-701.

37. Bergner, E., et al., *The period of untreated psychosis before treatment initiation: a qualitative study of family members' perspectives.* Compr Psychiatry, 2008. **49**(6): p. 530-6.

38. Morgan, C., et al., *Duration of untreated psychosis and ethnicity in the AESOP first-onset psychosis study.* Psychol Med, 2006. **36**(2): p. 239-47.

39. Allan, S., Oduola, S., Ricco, S., & Jodgekins, J., *"Jumping from place to place”: Service User Perspectives on Pathways to care in At-Risk Mental States and First Episode Psychosis.* Psychosis, under review.

40. Norman, R.M., et al., *Understanding delay in treatment for first-episode psychosis.* Psychol Med, 2004. **34**(2): p. 255-66.

41. Anderson, K., R. Fuhrer, and A. Malla, *The pathways to mental health care of first-episode psychosis patients: a systematic review.* Psychological medicine, 2010. **40**(10): p. 1585-1597.

42. Oduola, S., et al., *Change in incidence rates for psychosis in different ethnic groups in south London: findings from the Clinical Record Interactive Search-First Episode Psychosis (CRIS-FEP) study.* Psychol Med, 2019. **doi:10.1017/S0033291719003234**: p. 1-10.

43. Fearon, P., et al., *Incidence of schizophrenia and other psychoses in ethnic minority groups: results from the MRC AESOP Study.* Psycholological Medicine, 2006. **36**(11): p. 1541-50.

44. Oduola, S., Morgan, C. and Craig, TJK., *Are ethnic differences in pathways to care for psychosis in England reducing? An analysis of two population based studies of first episode psychosis in south London, UK.* , in *Early Intervention in Psychiatric Disorders Across Cultures*, A.V. Eric Chen., Dinesh Bhugra Editor. 2019, Oxford University Press: London.

45. Ventriglio, A., and Bhugra, D.,, *Early intervention in psychiatric disorders across cultures. First edition. Oxford: Oxford University Press*. Chen, E. Y., Ventriglio, A., & Bhugra, D. . 2019, Lodon: Oxford University Press.

46. Teuton, J., R. Bentall, and C. Dowrick, *Conceptualizing psychosis in Uganda: the perspective of indigenous and religious healers.* Transcult Psychiatry, 2007. **44**(1): p. 79-114.

47. Patel, V., *Explanatory models of mental illness in sub-Saharan Africa.* Social science & medicine, 1995. **40**(9): p. 1291-1298.

48. Kleinman, A., *Patients and healers in the context of culture: An exploration of the borderland between anthropology, medicine, and psychiatry*. Vol. 3. 1980: Univ of California Press.

49. Oduola, S. and J. Dykxhoorn, *Triple trauma, double uncertainty, and a singular imperative to address the mental health crises within asylum-seekers and refugees system: a commentary on Hvidtfeldt et al. (2021).* Soc Psychiatry Psychiatr Epidemiol, 2022. **57**(10): p. 2157-2159.

50. Patten, S.B., et al., *Perceived Stigma among Recipients of Mental Health Care in the General Canadian Population.* Canadian Journal of Psychiatry-Revue Canadienne De Psychiatrie, 2016. **61**(8): p. 480-488.

51. Memon, A., et al., *Perceived barriers to accessing mental health services among black and minority ethnic (BME) communities: a qualitative study in Southeast England.* BMJ Open, 2016. **6**(11): p. e012337.

52. Albert, N., et al., *Five years of specialised early intervention versus two years of specialised early intervention followed by three years of standard treatment for patients with a first episode psychosis: randomised, superiority, parallel group trial in Denmark (OPUS II).* Bmj, 2017. **356**: p. i6681.

53. Gafoor, R., et al., *Effect of early intervention on 5-year outcome in non-affective psychosis.* Br J Psychiatry, 2010. **196**(5): p. 372-6.

54. Hui, C.L.M., et al., *Effectiveness and optimal duration of early intervention treatment in adult-onset psychosis: a randomized clinical trial.* Psychol Med, 2022: p. 1-13.

55. Mustafa, S.S., et al., *Unfinished business: Functional outcomes in a randomized controlled trial of a three-year extension of early intervention versus regular care following two years of early intervention for psychosis.* Acta Psychiatr Scand, 2022. **145**(1): p. 86-99.

56. NHS England North, *Early Intervention in Psychosis: Guidance clarifying EIP and ARMS referral criteria*. 2016.

57. Yung, A.R. and P.D. McGorry, *The prodromal phase of first-episode psychosis: past and current conceptualizations.* Schizophr Bull, 1996. **22**(2): p. 353-70.

58. Sunwoo, M., et al., *Prevalence and outcomes of young people with concurrent autism spectrum disorder and first episode of psychosis.* Schizophr Res, 2020. **216**: p. 310-315.

59. Treise, C., et al., *Autism Spectrum Disorder in Early Intervention in Psychosis Services: Implementation and Findings of a 3-step Screening and Diagnostic Protocol.* J Psychiatr Pract, 2021. **27**(1): p. 23-32.

60. Ajnakina, O., et al., *Patterns of illness and care over the 5 years following onset of psychosis in different ethnic groups; the GAP-5 study.* Social Psychiatry and Psychiatric Epidemiology, 2017. **52**(9): p. 1101-1111.

61. Fusar-Poli, P., et al., *The psychosis high-risk state: a comprehensive state-of-the-art review.* JAMA Psychiatry, 2013. **70**(1): p. 107-20.

62. Clay, F., et al., *The over-35s: early intervention in psychosis services entering uncharted territory.* BJPsych Bull, 2018. **42**(4): p. 137-140.

63. Kreutzberg, A. and R. Jacobs, *Improving access to services for psychotic patients: does implementing a waiting time target make a difference.* Eur J Health Econ, 2020. **21**(5): p. 703-716.

64. Singh, K., et al., *Improving access to Early Intervention in Psychosis (EIP): the 2-week wait for cancer comes to psychosis.* BMJ Open Qual, 2018. **7**(3): p. e000190.

65. BMA. *Mental health workforce report*. 2021 [cited 2021 May 2021]; Available from: <https://www.bma.org.uk/advice-and-support/nhs-delivery-and-workforce/workforce/mental-health-workforce-report>.

AUTHOR CONTRIBUTIONS

SO, AM, CM and TC conceived the study. All authors were involved in the study design. Data collection was carried out by members of the CRIS-FEP research team. SO analysed data and drafted the manuscript. CM and TC supervised the study. EI and AM provided clinical advice and interpretations. All authors were involved in the interpretation of the data and in commenting on and revising drafts of the paper.

CONFLICT OF INTERESTS

CM is Editor in Chief, and SO is a member of the Editorial Board of Social Psychiatry and Psychiatric Epidemiology. They played no part in the decision of this manuscript. Other authors declare they have no conflict of interest

DATA SHARING

No additional data are available.
